# Maternal health behaviors during pregnancy in rural Northwestern China

**DOI:** 10.1186/s12884-020-03444-3

**Published:** 2020-11-30

**Authors:** Yue Ma, Yujuan Gao, Jason Li, Andrew Sun, Baozhu Wang, Jun Zhang, Sarah-Eve Dill, Alexis Medina, Scott Rozelle

**Affiliations:** 1 Rural Education Action Program, Freeman Spogli Institute for International Studies, Stanford University, Encina Hall, 616 Jane Stanford Way, Palo Alto, CA 94305 USA; 2 Harvard Medical School, Boston, MA 02115 USA; 3 Phillips Exeter Academy, 20 Main Street, Exeter, NH 03833 USA; 4 Northwest Women’s and Children’s Hospital, 1616 Yanxiang Road, Yanta District, Xi’an, 710061 Shaanxi China; 5 Shaanxi Xueqian Normal University, Shenhe 2nd Road, Chang’an, Xi’an, 710100 Shaanxi China; 6 Shaanxi Gender Development Solution, Room 5A, Shiji Guangchang Xingyuan, No.93 Heping Road, Xi’an, 710001 Shaanxi China

**Keywords:** Pregnant women, Health behaviors, Nutritional behaviors, Rural China

## Abstract

**Background:**

Maternal health during pregnancy is a key input in fetal health and child development. This study aims to systematically describe the health behaviors of pregnant women in rural China and identify which subgroups of women are more likely to engage in unhealthy behaviors during pregnancy.

**Methods:**

We surveyed 1088 pregnant women in rural northwestern China on exposure to unhealthy substances, nutritional behaviors, the timing and frequency of antenatal care, and demographic characteristics.

**Results:**

Pregnant women were active in seeking antenatal care and had low rates of alcohol consumption (5.1%), exposure to toxins (4.8%), and exposure to radiation (2.9%). However, tobacco exposure was widespread (40.3%), as was low dietary diversity (61.8%), unhealthy weight gain (59.7%), unhealthy pre-pregnancy BMI (29.7%), and no folic acid intake (17.1%). Maternal education is closely linked to better health behaviors, whereas experience with a previous pregnancy is not.

**Conclusions:**

Tobacco exposure and unhealthy nutritional behaviors are common among pregnant women in rural northwestern China. The findings indicate that in the absence of professional health information, relying on experience of previous pregnancies alone may not help rural women avoid unhealthy maternal behaviors. Maternal health education campaigns targeting nutrition and tobacco exposure during pregnancy may improve maternal, fetal, and child health in rural China.

## Background

Development in utero is crucial to the realization of a child’s future developmental potential [1]. Environmental and social conditions during pregnancy have been shown to affect infant morbidity, health, and developmental outcomes [2], which in turn affect health over the life course, as well as future economic participation and human capital accumulation [3]. Because of the consequential nature of this growth period, societal investment in promoting healthy fetal development has important implications for enhancing social and economic wellbeing [4].

While healthy fetal development depends on a number of factors, such as genetics and gestational period [5, 6], behaviors of the mother before and during pregnancy—including such behaviors as smoking, alcohol consumption and dietary choices— are key inputs in fetal development [7–9]. Unhealthy maternal behaviors during pregnancy can be classified into three broad categories: exposure to unhealthy substances, unhealthy nutritional behaviors, and inadequate antenatal care. To date, the literature has implicated unhealthy maternal behaviors in all three categories as causes of a wide range of adverse health outcomes in neonates, as well as permanent delays in physical and cognitive development later in life [10–14].

Studying fetal development and its link to the prenatal behavior of the mother is particularly important in rural China, where studies have found that large shares of infants and children are at risk for poor health and development [15–19]. In 2017, neonatal mortality (deaths during the first 28 days of life) in China accounted for half (49.5%) of all under-five deaths, with the neonatal mortality rate of rural areas double that of urban areas [15]. Developmental delays and micronutrient deficiencies also are prevalent in rural China. Multiple studies have found that nearly half of infants and toddlers across rural China are at risk for cognitive delay [16–18]. Additionally, a recent study found that roughly half of infants aged 6–12 months in rural areas of northwestern China are anemic [19]. The high prevalence of early health and developmental problems among young children indicates a need to better understand the factors contributing to unhealthy fetal, neonatal, and early childhood development.

While there are many sources of poor infant health and development [17, 20], poor maternal behaviors during pregnancy may be significant drivers of infant health issues in rural China. Although past studies have tended to focus on one or two behaviors, several studies have found high rates of individual unhealthy behaviors such as exposure to secondhand tobacco smoke, low or no intake of folic acid (an essential nutrient for fetal brain and spinal development), and poor nutrition [21, 22]. Additionally, according to the 2013 Fifth National Health Services Survey of China, almost half (43.9%) of pregnant women in rural areas of western China receive fewer than the recommended number of antenatal check-ups [23]. Such high rates of unhealthy behaviors during pregnancy suggest that a large share of children in rural China may have experienced sub-optimal fetal development due to substance exposure, inadequate nutrition, and insufficient antenatal care [7, 13, 24].

Although previous studies have filled an important need in describing unhealthy maternal behavior in rural China [25–27], there are several gaps in the literature to date. First, current studies are not comprehensive, focusing on only one or a few maternal behaviors and, in some analyses, without much detail or depth. For instance, the 2013 Fifth National Health Services Survey of China indicated that pregnant women had infrequent antenatal visits, but it did not specify how the average number of antenatal visits compares to national standards for maternal and child health [23]. Additionally, much of the existing research in rural China is out of date. The most recent study of pregnant women in rural China, conducted by Yang et al. (2017), used data from 2013 [14]. Due to the rapid nature of China’s economic development and advances in public health care over the past decade [28, 29], however, it is unclear whether these earlier studies are still representative of pregnant women in rural China today.

Finally, no study to date has systematically and empirically analyzed the demographic characteristics of pregnant women who engage in unhealthy behaviors in rural China. The international literature points to several potential risk factors for unhealthy maternal behaviors. For example, one study in the United States found that younger mothers tended to have lower quality diets [29, 30]. International studies have also shown that both low income and low maternal education are significantly associated with unhealthy maternal behaviors such as smoking, inadequate antenatal care, and low dietary quality [31, 32]. Understanding whether these and other factors similarly moderate the health behaviors of pregnant women in rural China can help public health researchers and practitioners target at-risk groups of women for interventions to promote healthy behavior and improve maternal and child health.

Economic and social characteristics unique to women and households in rural areas may also be linked to maternal health behaviors. Labor migration of rural women to urban areas, for example, may potentially correlate with health behaviors in several ways. Migration can significantly increase the family’s income [33], which is associated with healthier behavior. On the other hand, the working conditions of migrants (e.g., in factories) may be unhealthy for fetal development [34]. However, to date, little is known about the role of migrant experience in health behaviors among rural pregnant women in China.

Finally, the family structure of households in rural China may influence maternal health behavior. International literature suggests that family members such as husbands, mothers and mothers-in-law may influence the health behaviors of pregnant women [35, 36]. Because women in rural China traditionally move to their husbands’ homes after marriage [37], the influence of mothers-in-law may be especially strong. However, no study has yet examined examined the links between the husbands, mothers, or mothers-in-laws of pregnant women and their health behaviors such as exposure to toxins, nutrition, or antenatal care.

The overall goal of this study is to systematically describe the maternal health behaviors of pregnant women between 20 and 32 gestational weeks in rural areas of northwestern China. To meet this overall goal, we have two specific objectives. First, we describe maternal health behaviors in rural northwestern China, including exposure to unhealthy substances, nutritional behaviors, and the timing and frequency of antenatal care. Second, we analyze the correlations between unhealthy maternal behaviors and demographic characteristics to identify which subgroups of women are more likely to engage in unhealthy behaviors during pregnancy.

## Methods

### Study sample

We conducted our study in seven rural counties of a northwestern province in China. This province had a per capita GDP of around USD 8500 in 2017, ranking near the middle of China’s 31 provinces in terms of per capita GDP [38]. There are, however, a large number of nationally-designated poverty counties in the province. Of the seven counties in this study, five are nationally-designated poverty counties [39]. Therefore, in terms of economic development, our sample counties can be seen as representative of both moderate and relatively poor rural areas in northwestern China.

### Inclusion/exclusion criteria

From each of the seven sample counties, we recruited pregnant women from the local county-level Maternal and Child Hospitals (MCH). In order to be enrolled in this study, pregnant women had to meet four criteria: a.) the gestational age of the fetus must have been between 20 and 32 weeks; b.) the mother must have been visiting the MCH for a routine antenatal check-up at the time of enrollment; c.) the mother must have been a resident of the county with a rural residency permit (*hukou*); and d.) the mother must have had plans to live in a rural village or township within the county after delivery. We also excluded women who were living in the local county seat, which are typically wealthier and more urban than other areas within the county. All women meeting our criteria were enrolled in the study and administered a full survey during their hospital visit. In total, 1088 pregnant women from 210 townships were enrolled in the study.

### Data collection

Data were collected during the enrollment visit (described above), which occurred over two periods (each lasting 2 weeks) in November 2017 and April 2018. Surveys were conducted one-on-one by trained enumerators who read each survey question to the respondent, answered any questions the respondent had about the interpretation of the question, and then recorded the respondent’s answer on an electronic tablet.

The survey collected two main types of data: a.) individual and household characteristics; and b.) maternal health behaviors. Individual characteristics included the participant’s age; the gestational age of her fetus; her educational level; whether she was currently or had ever been a migrant worker; and whether this was her first pregnancy. Each participant was also asked questions about the characteristics of her household, including her husband’s age and level of education, as well as a listing of current household members (defined as individuals who had lived with the respondent for at least 3 months in the past year). Additionally, we constructed an index of family assets for each household using polychoral principal components analysis based on whether the family owned or had access to the following items: tap water, a toilet, a water heater, a washing machine, a computer, Internet, a refrigerator, an air conditioner, a motorcycle or electric bicycle, and a car [40].

In the second block of the survey, the respondents were asked questions about their health behaviors during pregnancy. Questions focused on three categories of maternal health behaviors that have been shown to affect fetal health and development: exposure to unhealthy substances, unhealthy nutritional behaviors, and inadequate antenatal care. To assess exposure to unhealthy substances, we asked respondents whether they had experienced the following during pregnancy: exposure to tobacco smoke (including secondhand smoke), alcohol consumption, exposure to toxins (e.g., pesticides, fumes in a factory), and exposure to radiation (e.g., X-rays). Following previous studies of substance exposure among pregnant women, we consider any exposure to a substance to be an unhealthy behavior [8, 41, 42]. A summary of the literature on these categories is presented in Table 5 in Appendix.

Questions aimed at understanding unhealthy nutritional behaviors focused on three variables: folic acid consumption, dietary diversity, pre-pregnancy BMI and weight gain during pregnancy. Folic acid consumption was assessed by self-report: women were asked whether or not they had consumed folic acid during their pregnancy. To measure dietary diversity, we used a food frequency questionnaire adapted from the Food and Agriculture Organization of the United Nations (FAO) [43] and the 2016 Dietary Guidelines for Chinese Residents [44, 45]. Each respondent was provided with a list of nine types of food and asked to indicate which food types she had consumed in the past 24 h. The nine food types include: 1) grains and cereals, 2) white tubers and roots, 3) fresh meat, 4) organ meat, 5) eggs, 6) milk and dairy products, 7) legumes (including soy and soy products), 8) vegetables, and 9) fruits. We calculated dietary diversity scores by summing the total number of food groups consumed by each participant. FAO guidelines do not set a cutoff score for dietary diversity and recommend that the mean dietary diversity score of the sample be used as a cutoff for analytical purposes; following these guidelines, women with dietary diversity scores below the mean score of the sample are considered to have low dietary diversity [43].

We also assessed the pre-pregnancy body mass index (BMI) and weight gain during pregnancy for each participant. Enumerators collected the self-reported height and weight before pregnancy of each sample respondent and measured the current weight and height of each participant using hospital equipment. We then calculated each participant’s BMI before pregnancy following World Health Organization (WHO) guidelines [46]. Participants were considered to be underweight if their BMI < 18.5; to have a healthy or normal weight if 18.5 ≤ BMI < 25; to be overweight if 25 ≤ BMI < 30; and to be obese if their BMI ≥ 30. We calculated weight gain during pregnancy for each participant by subtracting the weight of the participant before pregnancy from the observed weight during her visit. Recommendations by the WHO and the Institute of Medicine (IOM) indicate different standard weight gain ranges based on the gestational age of the fetus (Table 6 in Appendix) [47, 48]. Based on these guidelines, we separated the women into three groups: below the standard range of weight gain, within the standard range, and above the standard range.

The final sub-section of the survey collected information regarding antenatal care. Enumerators asked each pregnant woman to report the time of her first antenatal visit. Women were also asked to report the total number of antenatal visits to date. We then compared the reported number of antenatal visits to the Chinese Medical Association’s recommended antenatal visit schedule [48] to construct two variables. The first variable measured whether the first antenatal visit was early (before 6 weeks of gestational age), on time (between 6 and 13 weeks of gestational age), or late (after 13 weeks of gestational age). The second variable measured whether the total number of antenatal care visits to date was less than, equal to, or greater than the recommended number for the gestational age of the fetus.

### Statistical analysis

We first report descriptive statistics of maternal health behaviors, including exposure to unhealthy substances, unhealthy nutritional behaviors and antenatal care. We then examine the correlations between the individual and household characteristics of pregnant women and unhealthy maternal behaviors. To do so, we use a mixed-effects logistic regression model to conduct a series of multivariate regression analyses, each with one of our unhealthy behaviors as the dependent variable. For adequate statistical power and to avoid biasing the sample, this analysis only includes unhealthy behaviors prevalent among more than 10% of the sample, including low dietary diversity (1 = dietary diversity scores below the sample mean of 6; 0 = otherwise), weight gain out of standard range (1 = weight gain below or above WHO recommendations for gestational age of the fetus; 0 = otherwise), exposure to tobacco (1 = yes; 0 = no), BMI out of standard range (1 = BMI below 18.5 or above 25; 0 = BMI between 18.5–25) and no folic acid consumption (1 = did not consume folic acid; 0 = otherwise).

Based on the existing literature, we have included the following individual and household variables as potential confounders in the multivariate analyses: maternal age (whether the participant is older than the sample mean of 27 years), maternal education level, maternal migration history (both before and during pregnancy), whether this is the participant’s first pregnancy, husband’s age (whether her husband is older than the sample mean of 29 years), husband’s education, whether the participant lived with her husband for more than 3 months in the past year, whether the participant lived with her mother for more than 3 months in the past year, whether the participant had lived with her mother-in-law for more than 3 months in the past year, and family asset index. We also included county fixed effects in the model, as participants from different counties may be subject to county-specific policies or practices that may influence maternal health behaviors. Additionally, because data were collected in two separates waves (November 2017 and April 2018), we include time fixed effects. All statistical analyses were performed using STATA 14.1.

## Results

### Sample characteristics

Table 1 presents the basic socioeconomic and demographic characteristics of participants in the study. The average maternal age was 26.7 years, and the average gestational age was 26.3 weeks. About one third (33.4%) of pregnant women had completed senior high school or higher. The majority (87.1%) of women had worked as migrant workers in the past, and 29.4% were still migrant workers at the time of the survey. Women who were pregnant for the first time accounted for 40.3% of the participants. The average age of husbands of participants was 29.2 years, and 42.0% of husbands had completed senior high school or higher. More than half of our sample (57.3%) had lived with their husband for at least 3 months during the past year. Additionally, over two-thirds of the pregnant women in our sample (69.2%) had lived with their mothers or mothers-in-law for at least 3 months in the year prior to the survey.

**Table 1:** Summary statistics, *n* = 1088

Variables	Full Sample	
	Mean	Std. Dev.
***Individual characteristics***		
Age (years)	26.7	4.3
Gestational age (weeks)	26.3	3.6
Completed high school or above (1 = yes)	33.4	0.5
Currently working as a migrant (1 = yes)	29.4	0.5
Ever worked as a migrant (1 = yes)	87.1	0.3
First pregnancy (1 = yes)	40.3	0.5
***Household characteristics***		
Husband’s age (years)	29.2	4.6
Husband completed high school or above (1 = yes)	42.0	0.5
Lived with husband for > = 3 months (1 = yes)	57.3	0.5
Lived with mother / mother-in-law > = 3 (1 = yes) Months (1 = yes)	69.2	0.5
Asset index (PCA score)^a^	0.0	1.6

Source: Authors’ survey

^a^We constructed an index of family assets for each household using polychoral principal components analysis based on whether the family owned or had access to the following items: tap water, a toilet, a water heater, a washing machine, a computer, Internet, a refrigerator, an air conditioner, a motorcycle or electric bicycle, and a car

### Maternal health behaviors in rural China

Table 2 reports the rates of exposure to unhealthy substances and unhealthy nutritional behaviors in our sample. We found low rates of alcohol consumption (5.1%), exposure to toxins (4.8%), and exposure to radiation (2.9%) during pregnancy. However, tobacco exposure was widespread, with 40.3% of participants reporting exposure to tobacco smoke (including second-hand smoke) during pregnancy. The mean dietary diversity score was 6 food groups per day, and 61.8% had scores below the mean, indicating low dietary diversity. Nearly one-third (29.7%) of women were underweight or overweight at the start of their pregnancies, and 59.7% of pregnant women had gained either too much or too little weight during pregnancy given the gestational age of the fetus. Additionally, 17.1% of pregnant women were not taking folic acid during pregnancy.

**Table 2:** Health behaviors of pregnant women in rural China, *n* = 1088

Maternal Health Behaviors	(1)	(2)
	Frequency (n)	Percent (%)
[1] Have you smoked or been exposed to second-hand smoke during pregnancy?		
Yes	438	40.3
No	650	59.7
[2] Have you consumed alcohol during pregnancy?		
Yes	55	5.1
No	1033	94.9
[3] Have you been exposed to pesticides or other toxic chemicals during pregnancy?		
Yes	52	4.8
No	1036	95.2
[4] Have you been exposed to X-rays or other non-medical radiological substances during pregnancy?		
Yes	32	2.9
No	1056	97.1
[5] Have you taken folic acid during pregnancy?		
Yes	902	82.9
No	186	17.1
[6] Dietary diversity ^a^		
Higher dietary diversity (dietary diversity scores > = 6)	416	38.2
Low dietary diversity (dietary diversity scores < 6)	672	61.8
[7] BMI (Body Mass Index) before Pregnancy		
Underweight (BMI < 18.5)	195	17.9
Normal weight (BMI within 18.5 ~ 24.9)	765	70.3
Overweight (BMI within 25 ~ 29.9)	114	10.5
Obese (BMI > 30)	14	1.3
[8] Weight gain during pregnancy		
Below standard range	252	23.2
Within the standard range	439	40.3
Above standard range	397	36.5

Source: Authors’ survey

^a^ We use the mean dietary diversity score for the sample (6 out of 9) as the cutoff to define higher/lower dietary diversity

Uptake of antenatal care is shown in Table 3. Overall, women in our sample were relatively active in seeking antenatal care. More than half (61.2%) of pregnant women had their first antenatal visit within the recommended range of 6–13 gestational weeks, and about 30% had their first visit even earlier than recommended (before 6 weeks). Only 9.5% of pregnant women had their first antenatal visit later than recommended (after 13 weeks). We see a similar trend in the total number of antenatal visits: the majority (79.1%) of pregnant women had more than the recommended number of antenatal visits, while 15.3% of pregnant women had the recommended number of visits, and only 5.4% of pregnant women had fewer than the recommended number of antenatal visits.

**Table 3:** Timing and frequency of antenatal care visits, by gestational age (*n* = 1088)

	Pregnant Women with Different Gestational Age							
Antenatal care visits	20 ~ 24 weeks, *n* = 406		25 ~ 28 weeks, *n* = 321		29 ~ 32 weeks, *n* = 361		Total, *n* = 1088	
	Frequency (n)	Percent (%)	Frequency (n)	Percent (%)	Frequency (n)	Percent (%)	Frequency (n)	Percent (%)
[1] Time of first antenatal visit								
< 6 weeks	125	11.5	98	9.0	96	8.8	319	29.3
6–13 weeks	249	22.9	187	17.2	230	21.14	666	61.2
> 13 weeks	32	2.9	36	3.3	35	3.2	103	9.5
[2] Number of antenatal visits								
Less than recommended number	38	3.5	13	1.2	8	0.7	59	5.4
Equal to recommended number	101	9.3	46	4.2	20	1.8	167	15.3
More than recommended number	266	24.4	262	24.1	333	30.6	861	79.1

Source: Authors’ survey

### Characteristics associated with unhealthy maternal behaviors

Table 4 presents the correlations between unhealthy maternal behaviors and the demographic characteristics of participants. Because five of the unhealthy maternal behaviors were uncommon in our sample—alcohol consumption (5%), exposure to toxins (5%), exposure to radiation (3%), first antenatal visit later than recommended (9%), and fewer total antenatal visits than recommended (5%)—we excluded these from our multivariate analysis. Thus, of the ten unhealthy behaviors examined in this study, only five behaviors are included in the multivariate analyses: low dietary diversity, weight gain outside standard range, exposure to tobacco, BMI outside standard range, and no folic acid intake during pregnancy.

**Table 4:** Logistic regression between unhealthy maternal behaviors and demographic characteristics

	(1)		(2)		(3)		(4)		(5)		(6)		(7)	
VARIABLES	Low dietary diversity (1 = dietary diversity scores <= 6)		Weight gain out of standard range				Exposure to tobacco (including second-hand smoke)		BMI out of standard range				No folic acid consumption	
			(1 = below standard range)		(1 = above standard range)		(1 = exposure to tobacco)		(1 = below standard range)		(1 = above standard range)		(1 = no folic acid consumption)	
	**OR** ^**a**^	***P*** **Value**	**OR**	***P*** **Value**	**OR**	***P*** **Value**	**OR**	***P*** **Value**	**OR**	***P*** **Value**	**OR**	***P*** **Value**	**OR**	***P*** **Value**
	**(95% CI)**		**(95% CI)**		**(95% CI)**		**(95% CI)**		**(95% CI)**		**(95% CI)**		**(95% CI)**	
Age > mean	0.80	0.209	1.17	0.472	0.90	0.598	1.01	0.943	1.01	0.950	1.71**	0.044	0.90	0.649
	(0.57–1.13)		(0.76–1.79)		(0.62–1.32)		(0.72–1.42)		(0.64–1.61)		(1.02–2.87)		(0.58–1.40)	
Completed high school (or above)	0.68**	0.015	0.63**	0.031	0.84	0.322	1.36**	0.047	0.82	0.335	0.71	0.181	0.70	0.105
	(0.50–0.93)		(0.42–0.96)		(0.59–1.19)		(1.00–1.86)		(0.55–1.23)		(0.43–1.17)		(0.46–1.08)	
Currently working as migrant	0.98	0.872	1.17	0.436	1.06	0.718	0.68**	0.013	1.28	0.213	1.58*	0.050	0.76	0.204
	(0.72–1.33)		(0.79–1.73)		(0.76–1.49)		(0.50–0.92)		(0.87–1.89)		(1.00–2.50)		(0.50–1.16)	
Ever worked as migrant	1.14	0.496	0.90	0.668	0.88	0.546	0.88	0.515	1.15	0.605	0.79	0.428	0.82	0.426
	(0.78–1.68)		(0.54–1.48)		(0.57–1.34)		(0.60–1.29)		(0.68–1.94)		(0.45–1.41)		(0.51–1.33)	
First pregnancy	1.13	0.413	1.07	0.724	1.32*	0.090	1.18	0.263	1.04	0.815	1.24	0.353	0.85	0.417
	(0.84–1.51)		(0.74–1.54)		(0.96–1.82)		(0.89–1.56)		(0.73–1.50)		(0.79–1.95)		(0.58–1.25)	
Husband’s age > mean	1.33	0.109	0.56**	0.011	1.10	0.643	0.87	0.434	0.56**	0.014	0.88	0.644	1.08	0.747
	(0.94–1.89)		(0.36–0.88)		(0.74–1.61)		(0.62–1.23)		(0.35–0.89)		(0.52–1.50)		(0.69–1.68)	
Husband completed high school (or above)	0.81	0.149	1.06	0.752	1.18	0.325	1.01	0.967	1.18	0.397	0.87	0.551	0.89	0.564
	(0.60–1.08)		(0.72–1.57)		(0.85–1.63)		(0.75–1.35)		(0.81–1.71)		(0.54–1.38)		(0.60–1.32)	
Lived with husband for ≥3 months	1.20	0.198	0.80	0.230	0.75*	0.075	0.93	0.599	1.15	0.465	1.01	0.981	1.35	0.109
	(0.91–1.60)		(0.56–1.15)		(0.55–1.03)		(0.70–1.23)		(0.79–1.66)		(0.66–1.53)		(0.94–1.94)	
Lived with mother / mother-in-law ≥3 months	1.10	0.512	0.86	0.401	0.78	0.122	0.94	0.642	1.09	0.638	0.90	0.624	0.93	0.678
	(0.83–1.46)		(0.60–1.23)		(0.57–1.07)		(0.71–1.23)		(0.75–1.59)		(0.60–1.37)		(0.65–1.32)	
Asset index (PCA score)	0.82***	< 0.001	1.03	0.581	1.02	0.651	1.01	0.797	0.98	0.770	1.08	0.266	0.90*	0.055
	(0.75–0.90)		(0.92–1.15)		(0.93–1.12)		(0.93–1.10)		(0.88–1.10)		(0.94–1.23)		(0.81–1.00)	
County fixed effect	Yes		Yes		Yes		Yes		Yes		Yes		Yes	
Cohort fixed effect	Yes		Yes		Yes		Yes		Yes		Yes		Yes	
Observations	1088		691		836		1088		960		893		1088	

Source: Authors’ survey

^a^*Abbreviation*: *CI* 95% Confidence interval, reported in parentheses. Odds Ratio are showed in the table

* significant at 5%; ** significant at 5%; *** significant at 1%

We found age, education, and migrant status to be significantly associated with unhealthy maternal behaviors. First, older women had increased odds of having high BMI before pregnancy than younger women (*p* = 0.044). Second, women with higher levels of education had lower odds of having low dietary diversity during pregnancy (*p* = 0.015) and lower odds of having low weight gain during pregnancy (*p* = 0.031). Better educated women, however, had higher odds of being exposed to tobacco (including second-hand smoke) (*p* = 0.047). Finally, women who were still working as migrants during the time of the survey had lower odds of tobacco exposure (*p* = 0.013), but no such difference was found for women who had previously out-migrated but were now living in the local county. First-time pregnancy was not significantly associated with any of the unhealthy maternal behaviors measured.

Two household characteristics were also significantly associated with unhealthy nutritional behaviors (Table 4). First, women with younger husbands (≤ 29 years old) had greater odds of experiencing lower weight gain than recommended during pregnancy (*p* = 0.011) and greater odds of having low BMI before pregnancy (*p* = 0.014). Second, women from families with lower asset index values had greater odds of having low dietary diversity (*p* <  0.001). The educational level of husbands was not associated with any maternal health behaviors, and no significant differences in health behaviors were found among women who lived with their husbands, mothers, or mother-in-laws.

## Discussion

This study examined the health behaviors of pregnant women in rural China, drawing on data from 1088 pregnant women between 20 and 32 weeks of gestation. Using these data, we reported the prevalence of unhealthy maternal behaviors including exposure to toxic substances, nutritional behaviors, and antenatal care. We also identified individual and family characteristics associated with these behaviors.

According to the results, pregnant women were active in seeking antenatal care. Only 5.4% of pregnant women had less than the recommended number of antenatal visits, down from 25% in 2006 [27]. This suggests that a growing number of women recognize the importance of antenatal healthcare for fetal health. This may be because of recent government policies which have invested heavily in improving access to maternal and child healthcare in rural areas of western China through increased funding for Maternal and Child Hospitals, salary raises for medical staff, and subsidies and incentives for rural women to utilize the healthcare system [49]. However, 80% of pregnant women in our sample had more than the recommended number of antenatal visits, suggesting a possible overcorrection. This may be the result of physician-induced demand for services, a well-documented phenomenon in China [50].

Additionally, pregnant women generally avoided behaviors that are well known to be unhealthy, particularly exposure to unhealthy substances. According to the data, pregnant women in the sample had relatively low rates of alcohol consumption (5.1%), exposure to toxins (4.8%), and exposure to radiation (2.9%). Although the rate of alcohol consumption in our sample is higher than urban areas of China (0.7–1.6%) [51, 52], it is lower than that of developed countries like the United States (8.4%) [53]. The rate of exposure to toxins is lower than that found in both urban areas of China and in developed countries [54].

However, the data show high rates of tobacco exposure, with more than 40% of our sample reporting exposure to tobacco (including secondhand smoke) during pregnancy. Although we did not survey rates of active and passive smoking among our sample, previous studies have shown that in rural China, rates of smoking among men are much higher than among women (59% compared to 2%), suggesting that the majority of tobacco exposure in our sample may be due to secondhand smoke [21]. This is corroborated by past studies which have found that about 60% of rural mothers are exposed to secondhand smoke during pregnancy [26]. These findings suggest a lack of knowledge regarding the dangers of tobacco exposure during pregnancy, despite the substantial body of literature linking prenatal tobacco exposure to poor outcomes at birth and in childhood [7, 55, 56].

Unhealthy nutritional behaviors were also common among our sample. Most of the pregnant women surveyed had low dietary diversity (61.8%) and inadequate or excessive gestational weight gain (59.7%). A large share (29.7%) of women also had pre-pregnancy BMI outside of the recommended range. Additionally, 17.1% of pregnant women reported not taking any folic acid during pregnancy. Although this rate is lower than other prevalent unhealthy behaviors in our sample, it is higher than that of urban areas in the sample province, and it is also a indicative of a country-wide public health problem [57, 58]. This finding is also surprising given recent government policies providing free folic acid supplementation for pregnant women in rural areas [59]. One possible explanation may be that women in rural China do not know about the importance of folic acid intake during pregnancy or the availability of folic acid supplements. A previous study found that about 14% of rural pregnant women and mothers were unaware that folic acid should be taken during pregnancy, and almost 40% never used the free folic acid supplements provided by the government [57]. Educational campaigns to teach women about the importance of antenatal nutrition and micronutrient supplements may help to increase folic acid consumption and promote healthy nutritional behaviors among pregnant women in rural China.

When we examined the demographic characteristics associated with maternal health behaviors, we found that maternal education is closely linked to better health behaviors. In our sample, women with a high school education or above showed significantly greater odds of having low dietary diversity and low weight gain during pregnancy. This is consistent with previous studies, which have found that pregnant women with lower levels of education tend to have poorer-quality diets and increased risk of inadequate weight gain relative to women with higher levels of education [30, 60]. However, women with higher levels of education in our sample showed significantly greater odds of tobacco exposure during pregnancy, a finding that differs from previous studies, both in China and in other countries, which have linked higher education and income to reduced tobacco exposure [7, 31]. This discrepancy may be explained by the fact that previous studies have focused on women in more urban and more developed contexts than the rural areas from which we draw our sample; however, further research is needed to better understand this finding.

The results of our correlational analysis also show that experience with pregnancy was not linked to better health behaviors. There was no significant difference in the health behaviors of women who had been pregnant before and women who were pregnant for the first time. Similarly, living with mothers or mothers-in-law—who may be expected to provide guidance and information during pregnancy—was also not significantly associated with maternal health behaviors. However, when pregnant women had migrant experience during pregnancy, they had lower odds of tobacco exposure, possibly due to greater access to health education in cities. The findings indicate that in the absence of professional health information, relying on experience of previous pregnancies alone may not help rural women avoid unhealthy maternal behaviors.

From a policy perspective, high rates of at least five unhealthy maternal behaviors (tobacco exposure, low dietary diversity, too much/too little weight gain, unhealthy BMI, and no folic acid intake) suggest that large shares of women lack key information about the importance of maternal health, particularly nutrition, during pregnancy. Maternal health education campaigns targeting nutrition and tobacco exposure during pregnancy may therefore be necessary to improve maternal, fetal, and child health in rural China. Increasing maternal education is particularly important considering the high rates of birth defects (2.3%), low birth weight (9.3%) and growth retardation in children under 5 years old (11.2%) in this region [21, 61]. Providing increased information and guidance about maternal nutrition during antenatal visits, as well as expanding prenatal education classes to cover larger shares of rural women, may raise awareness of health and nutrition during pregnancy. In addition, public awareness campaigns around smoking, with a focus on husbands or other male friends and family members around pregnant women, may help to reduce the observed high rates of tobacco exposure and decrease neonatal mortality.

Our study makes significant contributions to the growing literature on health behaviors during pregnancy among women of different cultures and socioeconomic circumstances. This is the first study to describe the health behaviors of pregnant women in rural areas of western China, as well as the first study to systematically analyze individual and household characteristics that are associated with unhealthy behaviors. Additionally, by measuring the health behaviors of pregnant women during pregnancy rather than after delivery, our study reduces the possibility of recall error, which has been a limitation in the literature to date.

We also acknowledge three limitations to this study. First, because we collected data from pregnant women who were visiting their local county-level Maternal & Child Health hospital for antenatal care, our sample may not be entirely representative of rural pregnant women in northwestern China. Women from wealthier households may choose to travel further to municipal hospitals in provincial capitals or prefectural seats, while women from poorer households may go to smaller township hospitals or not seek antenatal care at all. Second, although we were able to reduce the possibility of recall bias by surveying women during pregnancy rather than after delivery, we still relied heavily upon participant self-report, which means that we cannot entirely rule out the possibility of recall error. Finally, although we collected detailed information on a number of health behaviors, we did not collect data from pregnant women regarding all aspects of their lifestyles. For example, we do not have information on their physical activity levels. Future studies should incorporate both interview and observational survey data to better assess maternal health behaviors during pregnancy.

## Conclusion

Maternal health behaviors during pregnancy are key inputs in fetal health and development. Our study shows that although women in rural China are receiving adequate antenatal care through the public healthcare system, inadequate nutrition and exposure to tobacco remain prevalent and may have significant consequences for fetal health. Improving maternal nutrition and reducing tobacco exposure during pregnancy should therefore be a priority for health care reforms in rural China. Given the significant relationship between maternal education and health behaviors, policy makers and practitioners should develop maternal health education campaigns, focusing on nutrition and tobacco exposure during pregnancy, to improve maternal and fetal health in utero.

## Data Availability

The datasets used and/or analyzed during the current study are available from the corresponding author on reasonable request.

## Appendix

**Table 5:** Literature on Unhealthy Maternal Behaviors and its Influence on Fetal and Child Development

Category	Unhealthy maternal behaviors	Influence on child development	Author
Exposures to unhealthy substances	Exposure to tobacco (including second-hand smoke)	Increases child’s risk for asthma and Attention-Deficit/Hyperactivity Disorder (ADHD)	He [26] Román [55] Hernández-Martínez [7] Rodriguez [62]
	Alcohol consumption	Affects child brain development, damages selective brain structures, and increases the risk of growth retardation; Mothers who consumed alcohol during pregnancy were also at elevated risk of experiencing placental abruption	Aliyu [8] Jones [63] Isaksen [64] Nilsson [65]
	Exposure to toxins, such as pesticides	Negatively impacts birth weight and brain development	Bouchard [41] Currie [66]
	Exposure to radiation	Associated with prenatal death, intrauterine growth restriction, smaller head size, intellectual disability, organ malformation, and childhood cancer	Williams [42]
Inadequate maternal nutrition	Inadequate folic acid consumption in early pregnancy	Increases risk for neural tube defects	McStay [67] Yang [22]
	Out of standard range for pre-pregnancy maternal Body Mass Index (BMI) and pregnancy weight gain	Associated with higher risks of low birth weight and preterm birth and increased risk of cardiovascular, metabolic and neurological disorders later in life	Pan [25] Rivera [62] Diouf [68] Stang [69]
	Low dietary diversity	Increased risk of low birth weight and preterm birth	Yang [22] Zerfu [9]
Inadequate antenatal care	Less than the recommended number of antenatal visits	Increased risk of intrauterine growth retardation and low birth weight	Vogel [24]
	Later than recommended first antenatal visit	Increased risk of low birth weight and infant mortality	Khanal [70]

**Table 6:** Recommended Pregnancy Weight Gain Rates by Pre-pregnancy BMI

	Rates of Weight Gain^a^ 2nd and 3rd Trimester
Pre-pregnancy BMI	Mean (range) in kg/week
Underweight (< 18.5 kg/m^2^)	0.51 (0.44–0.58)
Normal weight (18.5–24.9 kg/m^2^)	0.42 (0.35–0.50)
Overweight (25.0–29.9 kg/m^2^)	0.28 (0.23–0.33)
Obese (≥ 30.0 kg/m^2^)	0.22 (0.17–0.27)

Modified from Institute of Medicine (US). Weight gain during pregnancy: reexamining the guidelines. Washington, DC. National Academies Press; 2009.©2009 National Academy of Sciences

^a^Calculations assume a 0.5–2 kg weight gain in the first trimester

## Abbreviations

WHO: World Health Organization
UNICEF: The United Nations Children’s Fund
NHFPC: National Health and Family Planning Commission of the People’s Republic of China
UNESCO: United Nations Educational, Scientific and Cultural Organization
GDP: Gross domestic product
FAO: Food and Agriculture Organization
BMI: Body Mass Index
IOM: Institute of Medicine
